# Pre-Slaughter Stress Affects Ryanodine Receptor Protein Gene Expression and the Water-Holding Capacity in Fillets of the Nile Tilapia

**DOI:** 10.1371/journal.pone.0129145

**Published:** 2015-06-08

**Authors:** Elenice S. R. Goes, Jorge A. F. Lara, Eliane Gasparino, Ana P. Del Vesco, Marcio D. Goes, Luiz Alexandre Filho, Ricardo P. Ribeiro

**Affiliations:** 1 Universidade Estadual de Maringá–UEM–Maringá, Paraná, 87020–900, Brazil; 2 Empresa Brasileira de Pesquisa Agropecuária–EMBRAPA Pantanal–Corumbá, Mato Grosso do Sul, Brazil; 3 Department of Animal Science, Universidade Estadual de Maringá–UEM–Maringá, Paraná, Brazil; 4 Universidade Federal do Paraná–UFPR–Palotina, Paraná, Brazil; Institute of Hydrobiology, Chinese Academy of Sciences, CHINA

## Abstract

Current study evaluated the effect of pre-slaughter stress on serum cortisol levels, pH, colorimetry, water-holding capacity (WHC) and gene expression of ryanodine receptors (RyR1 and RyR3) in the Nile tilapia. A 3x4 factorial scheme experiment was conducted comprising three densities (100, 200, 400 kg/m³) with four transportation times (60, 120, 180, and 240 minutes).Transportation times alone reduced cortisol levels up to 180 minutes, followed by increased WHC and mRNA expression, RyR1 and RyR3 (200 kg/m³ density). No effect of density x transportation time interacted on the evaluated parameters. Results provided the first evidence that pre-slaughter stress affected ryanodine gene expression receptors and, consequently, the water-holding capacity in tilapia fillets.

## Introduction

Fish management procedures in aquaculture, such as capture, handling and transportation of fish, are often traumatic, with serious physiological and biochemical reactions. Transporting live fish is a multiple-phase operation that should be managed to minimize stress and costs involved. The transport of fish in tank trucks requires special care to ensure that water quality parameters (especially temperature and oxygen content) and fish density requirements are maintained [1].

Decrease in pre-slaughter stress is important since vigorous swimming causes intense usage of the white muscle, a rise in anaerobic glycolysis and lactic acid production, with a decrease in the muscle´s pH [2, 3, 4]. Further, a muscle protein structural degradation occurs which reduces the meat water-holding capacity [5]. Furthermore, since the weight of meat is reduced, the lost exudate contains significant amounts of protein, mainly sarcoplasmic proteins. Liabilities of millions of dollars occur yearly due to meat with low water retention [6].

Acidification of muscle due to lactic acid level causes animals that struggle at slaughter go into rigor mortis very rapidly [7]. Fish processed during rigor mortis has impaired flesh quality [7] and lower fillet yield [8].

Pre-slaughter stress may be one of the reasons for pale, soft and exudative (PSE) meat development in swine and poultry. The above-mentioned types of meat have a pale color, flaccid texture and a low water-holding capacity, which makes them exudative. Although the mechanisms involved in PSE meat development are not fully understood, it is generally thought that abnormally rapid postmortem skeletal muscle metabolism causes PSE meats [9]. The condition is associated with an excess of calcium ions (Ca^2+^) in the cytosol [10].

Release control of intracellular Ca^2+^ is accomplished through the ryanodine receptors (RyR) which are large homotetrametric protein complexes [11]. Contraction in the muscle is due to neurostimulation through the motor endplate which releases calcium from the sarcoplasmic reticulum to the sarcoplasm. The physiological consequences of Ca^2+^ level increase prior to slaughter include a rise in muscle hypermetabolism which leads to postmortem warming and acidosis in the muscle tissue [12].

Three homologous isoforms of ryanodine receptors were identified in non-mammalian tissues based on molecular, immunological, biochemical, and physiological results [13,14]. Adult mammals predominantly express RyR1 isoform in the skeletal muscle, while non-mammalians, including fish, express the two isoforms, RyR1 and RyR3 [15]. The RyR2 isoform is expressed primarily in the cardiac tissue of teleost fish [11]. The two RyR isoforms (1 and 3) are encoded by different genes [9] and are present in similar quantities in the muscle [13].

Due to the importance of the water-holding capacity in the fish industry, the genetic causes that affect this parameter were assessed. Studies on fish that link skeletal muscle RyR expression with ante-mortem acute stress and meat quality are not yet extant. Current analysis evaluated transport-provoked stress and its influence on water-holding capacity and RyR gene expression in Nile tilapia (*Orechromis niloticus*).

## Materials and Methods

Method was carried out in accordance with the guidelines of the Brazilian College for Animal Experimentation (COBEA; http://www.cobea.org.br) and was approved by the Committee for Ethics on the Use of Animals in Research (CEAE) of the Universidade Estadual de Maringá, Maringá PR Brazil (Protocol 007/2013-CEAE, March 2013).

### Animals

The fish were grown in net-tanks located in the Rio do Corvo, municipality of Diamante do Norte PR Brazil (22°39’ S; 52°46’ W). Further, 1,600 juvenile tilapias were sexually reversed with 17-α-methyl-testosterone masculinizing hormone provided in the diet during the larval stage. The fish were derived from the sixth generation of the Genetic Improvement of Nile tilapia Program, GIFT/TILAMAX variety, from the Universidade Estadual de Maringá. The animals had a mean weight 69.75±9.92 g and were conditioned at a 9.3 kg/m^3^ average initial density in two 6 m^3^ net-tanks. The fish were fed three times a day on a commercial ration containing 32% crude protein and 3500 Kcal/kg digestible energy, during 210 days, between March and October 2013. Prior to the experiment, the animals fasted for 24h to empty the digestive tract.

### Experimental Design

An experiment with different density and transportation times was performed to simulate different conditions and thus differentiate levels of ante-mortem acute stress in tilapias. The experiment was conducted in a 3x4 factorial scheme, comprising three densities (100, 200, and 400 live weight kg /m^3^) and four transportation times (60, 120, 180 and 240 minutes) and a control treatment (fish removed from the tanks and immediately euthanized). There was a total of 13 treatments, with 10 repetitions per treatment (the fish was the experimental unit), in a total of 130 fish (866.86±143.98 g average weight and 33.80±1.63 cm overall length).

Three 1000-L fiberglass transportation boxes, equipped with diffusers and oxygen cylinders, were used to transport the live fish. Temperature and dissolved oxygen were tracked during transportation by a portable apparatus (YSI Model 55 Dissolved oxygen, YSI Incorporated, Yellow Springs, USA) to maintain a 25.44±1.29°C average temperature and 8.05±3.19 mg/L average dissolved oxygen. Further, 6 mg/L of sodium chloride were added to the water.

Initially, the fish were randomly removed from the net-tanks with a scoop net; they were weighed on a portable scale and placed into three transportation boxes. Each box contained a different density (100, 200 and 400 kg of fish/m^3^). The boxes were placed on a truck and transported at different time marks (60, 120, 180, and 240 min); at the end of each time point, transportation was stopped and 10 animals were removed from each density treatment.

The fish were transported in local roads at speeds mimicking commuting between harvest and processing unit (around 30 mph). The samples were taken in a processing facility, and transport times (60, 120, 180 and 240 min) were coordinated with the arrival of the truck to the processing unit. Each sample lasted for about five minutes, and during this time the transport truck stood still.

Fish from each treatment were removed from the boxes and anesthetized with benzocaine at a proportion of 1 g/10mL alcohol/10L water; blood was then collected from five fish by caudal puncture for cortisol analysis and muscle collection for gene expression. The fish were euthanized by dissection of the spinal cord, conditioned in water and ice, and their ventral abdominal cavity was opened from the urogenital orifice up to the jaw bones, followed by the careful removal of the viscera. The fish were beheaded, skin was removed and filleting was done manually. The whole skinless fillets with fillet parings (V-cut fillets) were washed in 5 ppm chlorinated water, packed and refrigerated (4°C) until analysis.

### Cortisol analysis

Two milliliters of blood from each fish (n = 5) were collected by caudal puncture with disposable syringes and serum was obtained after immersing blood samples in a warm water bath at 37°C for 10 minutes, followed by centrifugation at 1000 xg for 10 minutes and collection of the supernatant (serum). The analysis of serum cortisol concentration was performed by chemiluminescence microparticle immunoassay (CMIA) with Architect Ci8200 (Abbott Laboratories, Abbott Park, Ill, USA) with reagents of the same brand.

### pH, color and water-holding capacity

Indicators of fillets quality were evaluated by pH, color and water-holding capacity (WHC). Color and pH were analyzed in refrigerated samples, while WHC was performed from frozen fillets. Further, pH was measured at 36 hours post-mortem by direct insertion in the muscle of pH-meter Toledo Mettler (model 1140, São Paulo, Brazil), linked to a glass electrode Mettler Toledo MG.DXK.

The color of the fillets was determined 36 hours post-mortem with a direct color reader (MINOLTA model CR-10; Minolta Camera Co., Osaka, Japan) to obtain the color rates: L*(brightness/darkness) and b* (yellowness/blueness). Measurements were taken from the ventral face of the fillet, at six different places, and averaged.

For WHC analysis, fillet samples (n = 10) were defrosted and weighed with an analytical scale (Shimadzu AUY-220, Shimadzu Corporation, Kyoto, Japan); 1 g of raw fillet, in triplicate, were placed in 1.5 mL tubes with filter paper. The tubes where centrifuged at 1318 xg for 4 minutes at 4°C in an Eppendorf centrifuge (model 5430R, Eppendorf North America, New York, USA). The samples were weighed after centrifugation and dried in a Fanem buffer (model Orion 515, Fanem, São Paulo, Brazil) at 70°C for 12 hours. The dry samples were weighed once more and the equation below (1) was used to calculate WHC:

$$WHC\%=\frac{PCSW-DSW}{ISW}\times 100$$ (1)

where WHC% = Water-Holding Capacity; ISW = Initial Sample Weight; PCSW = Post-Centrifugation Sample Weight; DSW = Dry Sample Weight.

### Gene Expression

Approximately 2 g of white muscle from 5 fish were collected from each treatment to evaluate RyR1 and RyR3 gene expression in the different treatments. The muscle samples were stored in an RNA Holder (BioAgency Biotecnologia, Brazil) at -20°C until RNA extraction. Total RNA was extracted with a Trizol reagent (Invitrogen, Carlsbad CA, USA) following manufacturer´s instructions, at a proportion of 1 mL per 100 mg of tissue. All materials used were previously treated with RNase inhibitor—RNase AWAY (Invitrogen, Carlsbad, CA, USA). Samples were measured by a spectrophotometer (Picodrop Limited, Hinxton, UK) to evaluate total RNA concentration. The RNA integrity was evaluated in 1% agar gel colored with SYBR Safe DNA Gel Stain (Invitrogen, Carlsbad CA, USA) and viewed in a transilluminator with ultraviolet light. RNA samples were treated with DNase I (Invitrogen, Carlsbad, CA, USA) for possible genomic DNA waste removal, according to manufacturer´s recommendations. Further, cDNA was performed with SuperScrippt III First-Strand Synthesis Super Mix kit (Invitrogen, Carlsbad CA, USA), following manufacturer´s instructions. Samples were stored at -20°C until use.

For real time PCR reactions, the fluorescent dye Platinum SYBR Green qPCR SuperMix-UDG with ROX (Invitrogen, Carlsbad CA, USA) was used, following manufacturer´s instructions. All analyses were performed in a 25 μL-glass, in duplicates. The reactions were conducted in strips in a BIORAD iQ5 iCycler (Bio-Rad Laboratories, Inc. Hercules, CA, USA) appliance.

The primers in the reactions followed sequences at site www.ncbi.nlm.nih.gov for type 1 ryanodine receptor protein–RyR1 (XM_005474818.1) gene and type 3 ryanodine receptor protein—RyR3 (XM_005453445) for *Oreochromis niloticus* was determined according to site www.idtdna.com. RyR1 primers provided sequences F: TTCTACCAACACCCCAATCTG and R: AGTAACACAGGAAACGACAGC, with amplicon size 143 bp, whereas RyR3 primers provided sequences F: TGTTTCATCTGTGGGATCGG and R: GTGTGCTCTGTCTCATCCTTG, with amplicon size 140 bp. The expression levels of the Nile tilapia´s RyR1 and RyR3 genes were calculated according to the expression of ß-actin housekeeping gene (XM_003455949) by primer developed by Yang et al. [16] (F: TGGTGGGTATGGGTCAGAAAG and R: CTGTTGGCTTTGGGGTTCA, amplicon size 217 bp).

The primers for genes (RyR1 and RyR3) analyzed in current study proved to be adequate for real-time PCR analysis. Amplification efficiency was similar to the genes which ranged between 90 and 110%. Analysis of the dissociation curve revealed neither the presence of unspecific products nor the formation of dimers of the primers. The above demonstrates the reliability of data in the mRNA expression of the studied genes. The statistical analysis of β-actin as endogenous control did not reveal any statistically significant difference between the treatments and proved their efficiency as an endogenous control.

### Statistical analysis

The 2^ΔCT^ method was used for the relative quantification analysis, with data in arbitrary units. Data obtained in the cortisol analyses, pH, colorimetry, water-holding capacity, and RyR1 and RyR3 gene expression were submitted to variance analysis by GLM procedure from the statistical computer program Statistical Analysis System (SAS, SAS Inst. Inc. Cary, NC, USA). Density effect, transportation time and interaction among factor were evaluated. When a significant difference (P<0.05) was noted in the density factor, Tukey's test was applied, while the time factor was submitted to a regression analysis at 5% significance. Treatments were also compared with control treatment by Dunnet's test. The results are expressed as averages and standard error of the mean.

## Results

### Stress characterization

Cortisol serum levels were evaluated to characterize stress. Transportation density did not have any effect (P = 0.6229), with averages 20.84±1.93, 22.47±2.19 and 23.36±2.95 μg/dL respectively for densities 100, 200 and 400 kg/m^3^. Similarly, the interaction between density and transportation time did not have any effect (P = 0.2204) on cortisol serum levels.

**Fig 1:**
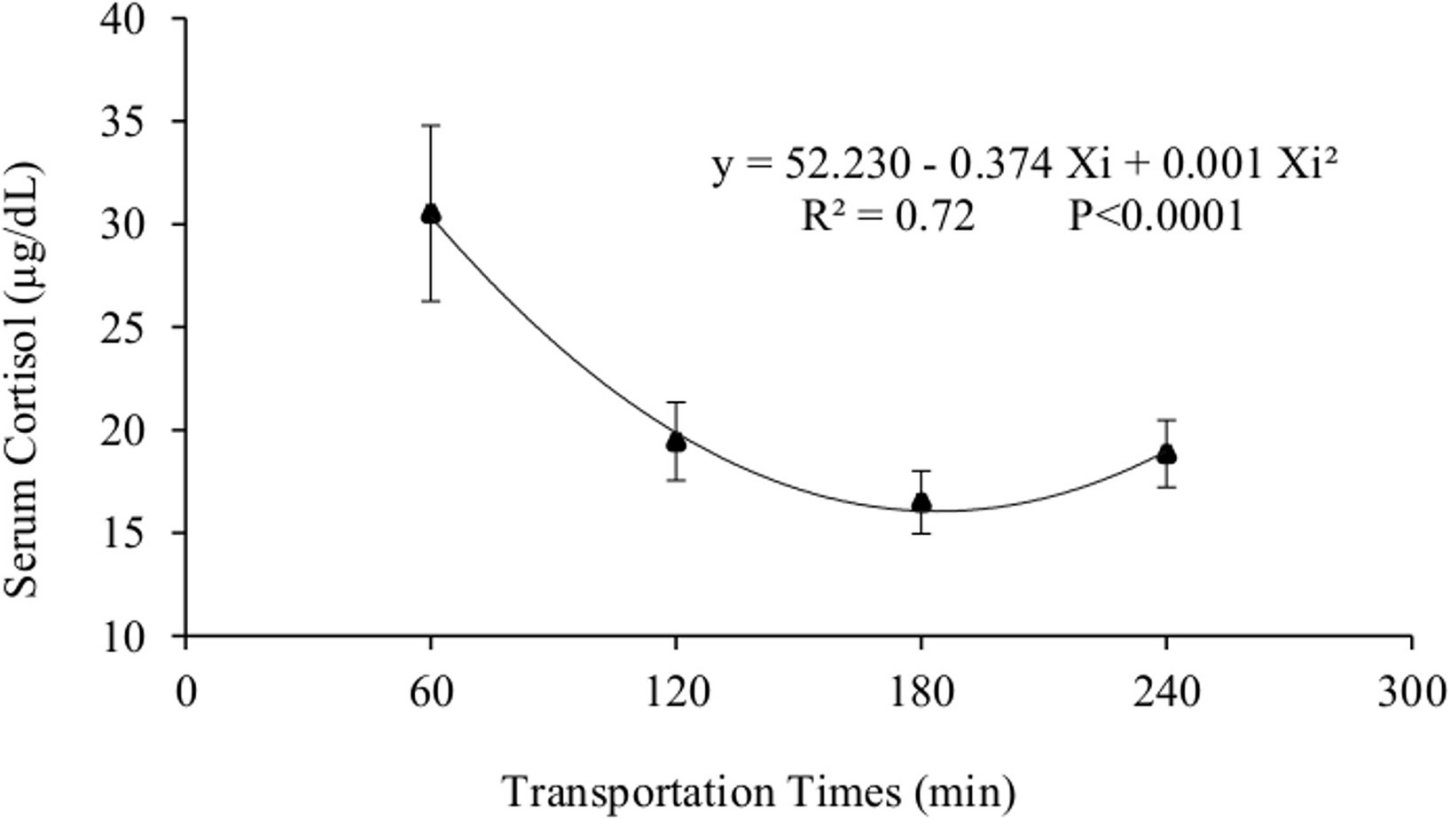
Stress characterization. Serum cortisol averages from Nile tilapia submitted to different transportation times (60, 120, 180, and 240 minutes). Vertical lines represent the standard error of the mean between the fifteen replications per time of transportation.

Transportation time had a significant effect since the quadratic regression (P = 0.0001) had the best adjustment to the data (equation y = 52.230–0.374 Xi + 0.001 Xi^2^, R^2^ = 0.72) (Fig 1). Highest cortisol level (30.52 μg/dL) occurred after 60 minutes of transportation and the lowest cortisol level (16.49 μg/dL) occurred after 180 minutes. When treatment and control cortisol levels were compared, only treatment 400 kg/m^3^ + 60 min had a different average (41.02 μg/dL) from control (P<0.01) (13.34 μg/dL).

### pH, color and water-holding capacity

The interaction between density and transportation time did not significantly affect (P>0.05) the pH and color (L * and b *) parameters of the fillets (Table 1). Transportation time showed no significant regression (P>0.05) when independently analyzed. Isolating the density factor, there was a significant effect only for chroma b * (P = 0.0359), a higher mean (8.05) for density 400 kg/m^3^. In the case of pH, when comparing treatment averages with control, it was observed that treatments 100 kg/m^3^ + 120 min and 400 kg/m^3^ + 180 min had average pH (6.20 and 6.20 respectively) significantly higher (P<0.05) than the control treatment mean (5.85).

**Table 1:** pH and colour values of Nile tilapia fillets submitted to different transportation densities (100, 200, and 400 kg/m^3^) and different transportation times (60, 120, 180, and 240 minutes).

Densities (kg/m^3^)	Time (min)	pH	L*	b*^1^
**100**	60	6.16±0.06	46.10±0.24	8.09±0.15
	120	6.20±0.09*	44.38±0.65	7.25±0.14
	180	6.04±0.06	43.90±0.45	7.57±0.25
	240	6.09±0.06	45.23±0.57	7.62±0.32
**200**	60	6.00±0.11	44.99±0.35	7.97±0.29
	120	6.05±0.13	45.29±0.35	7.29±0.22
	180	6.07±0.08	45.07±0.41	7.78±0.29
	240	6.11±0.06	43.88±0.42	7.55±0.23
**400**	60	6.04±0.06	45.64±0.44	8.20±0.24
	120	5.91±0.06	45.04±0.53	8.20±0.28
	180	6.20±0.06*	45.32±0.65	7.83±0.32
	240	6.12±0.04	44.77±0.42	7.96±0.24
**Control**		5.85±0.12	45.08±0.51^NS^	8.16±0.21^NS^
**Densities (kg/m^3^)**				
**100**		6.12±0.03	44.87±0.27	7.63±0.12B
**200**		6.06±0.04	44.80±0.20	7.64±0.13B
**400**		6.07±0.04	45.19±0.25	8.05±0.13A
**Times (minutes)**				
**60**		6.07± 0.04	45.58±0.21	8.08±0.13
**120**		6.05±0.06	44.90±0.30	7.58±0.15
**180**		6.10±0.04	44.76±0.31	7.73±0.16
**240**		6.11 ±0.03	44.63±0.28	7.71±0.15
**Densities x times effect**		0.1155	0.0555	0.6071
**Densities effect**		0.4342	0.485	0.0359^a^
**Times effect**		0.7773	0.0711	0.0949

^1^L*: brightness/darkness; b*: yellowness/blueness

* indicates a difference in treatments from the control based on Dunnett's test

^a,B^ Means in the same column followed by different letters differ by Tukey’s test^.^

Values are expressed as the mean ± standard error of the mean (n = 10).

NS = Non-significant

**Fig 2:**
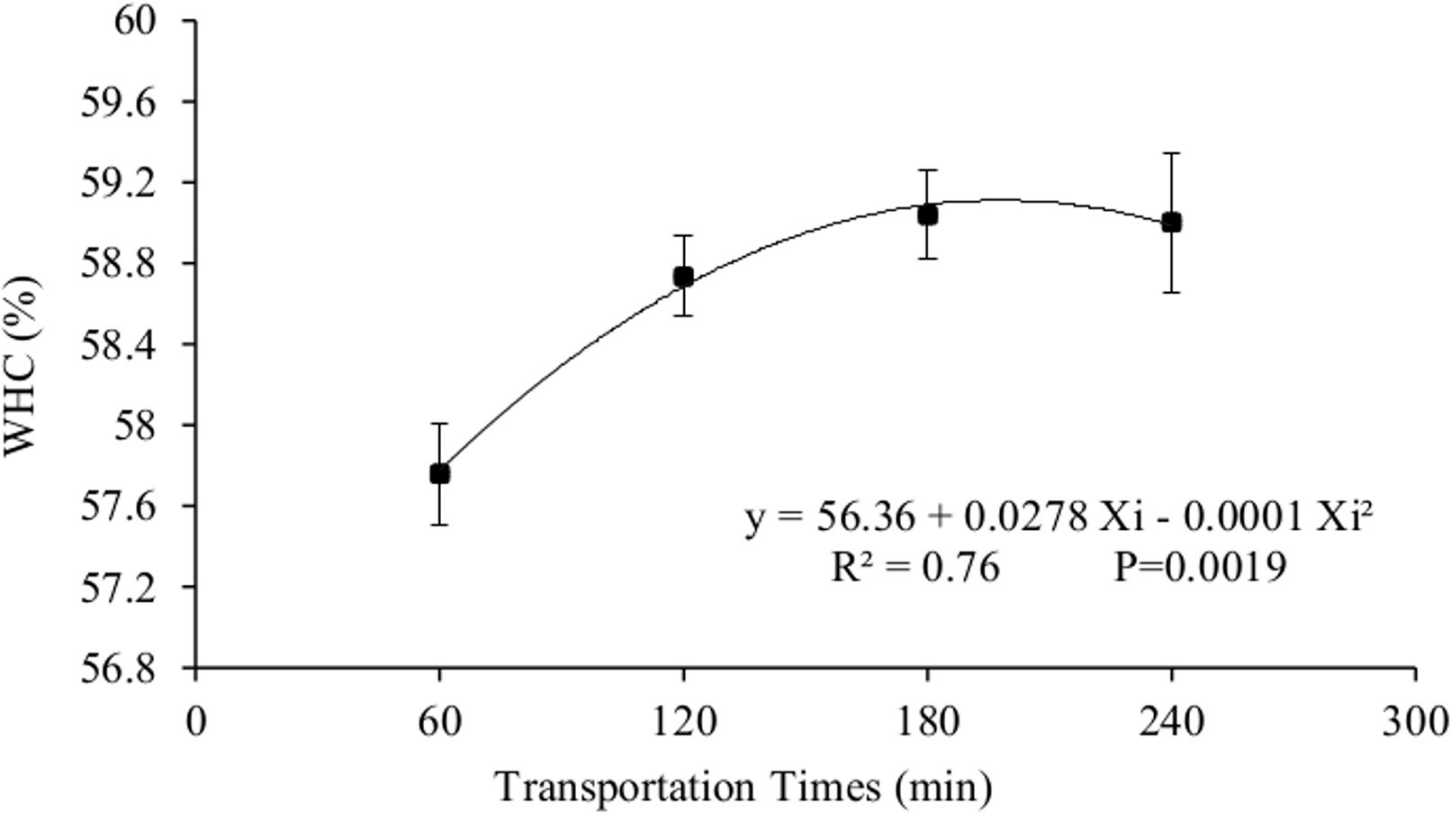
Water Holding Capacity of fillets. Water Holding Capacity (WHC%) in Nile tilapia fillets submitted to different transportation times (60, 120, 180, and 240 minutes). Vertical lines represent the standard error of the mean between the thirty replications per time of transportation.

In the case of water-holding capacity (WHC), only transportation time had a significant effect (P = 0.0019), as the quadratic regression equation was the best fit for the data (y = 56.36 + 0.0278Xi– 0.0001Xi^2^, R^2^ = 0.76) (Fig 2). WHC increased as time increased, until 180 minutes, which had the maximum of 59.04% of WHC.

In comparison to control treatments, treatments 200 kg/m^3^ + 60 min and 200 kg/m^3^ + 240 min had different averages (57.19% and 59.64% respectively) from the control (58.33%) by Dunnett's test (P = 0.0235).

### Ryanodine receptor protein gene expression

**Fig 3:**
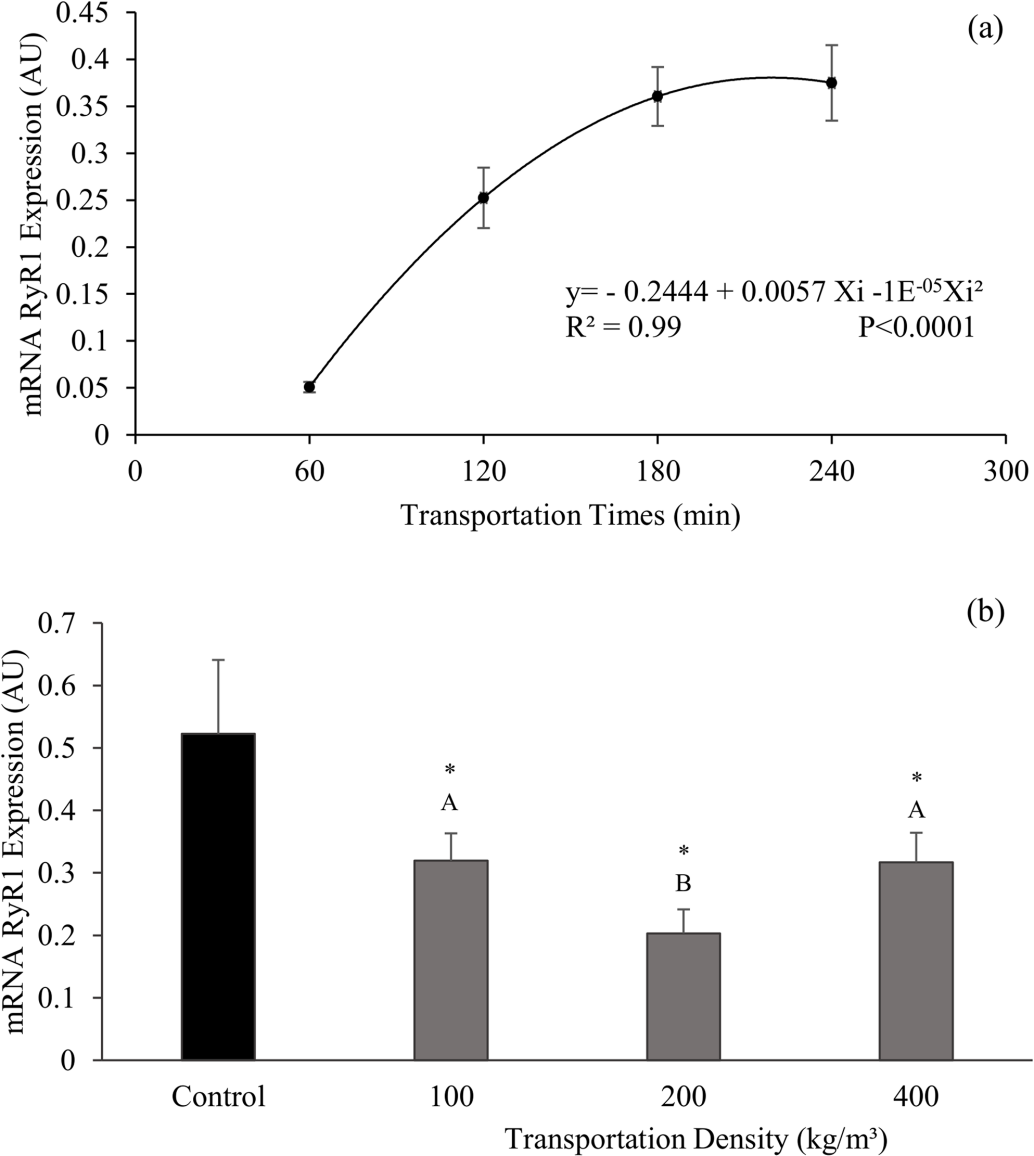
Protein receptor ryanodine type 1 gene expression in muscle. Nile tilapia mRNA RyR1 expression submitted to different transportation times (60, 120, 180, and 240 minutes) (a) and different transportation densities (100, 200, and 400 kg/m^3^) (b). Different letters show significant differences between treatments by Tukey's Test (P = 0.0044). (*) indicates a difference in treatments from the control based on Dunnett's test (P<0.05). AU is abbreviation for Arbitrary Unit. In Fig 3a, vertical lines represent the standard error of the mean between the fifteen replications per time of transportation; and in Fig 3b. vertical bars represent the standard error of the mean between the twenty replications per transportation density (control is n = 5).

For type 1 ryanodine receptor protein (RyR1) gene expression, there was no significant difference in the interaction between density and transportation time (P = 0.2424). However, the isolation of time and density factors was significant (P<0.01). In the case of transportation time (Fig 3A), a quadratic regression occurred (y = -0.2444 + 0.0057Xi -1E^-05^Xi^2^, R^2^ = 0.99) for mRNA RyR1 expression in which 60 min time showed the lowest expression level (0.036 AU), with an increase until 240 min (0.400 AU). When different transportation times were compared with control treatment by Dunnett's test, there was a significant difference (P<0.0001) between average times 60, 120, and 180 min. In fact, they demonstrated a lower expression of RyR1 mRNA when compared to control (0.523 AU).

There was a difference (P = 0.0044) in mRNA RyR1 in the isolation of the transport density factor. Average density (200 kg/m^3^) showed a lower expression (0.203 AU) when compared to the other densities (Fig 3B). When densities are compared to control treatment, a significant difference (P = 0.0004) was noted. Densities had a mean lower expression when compared to control.

**Fig 4:**
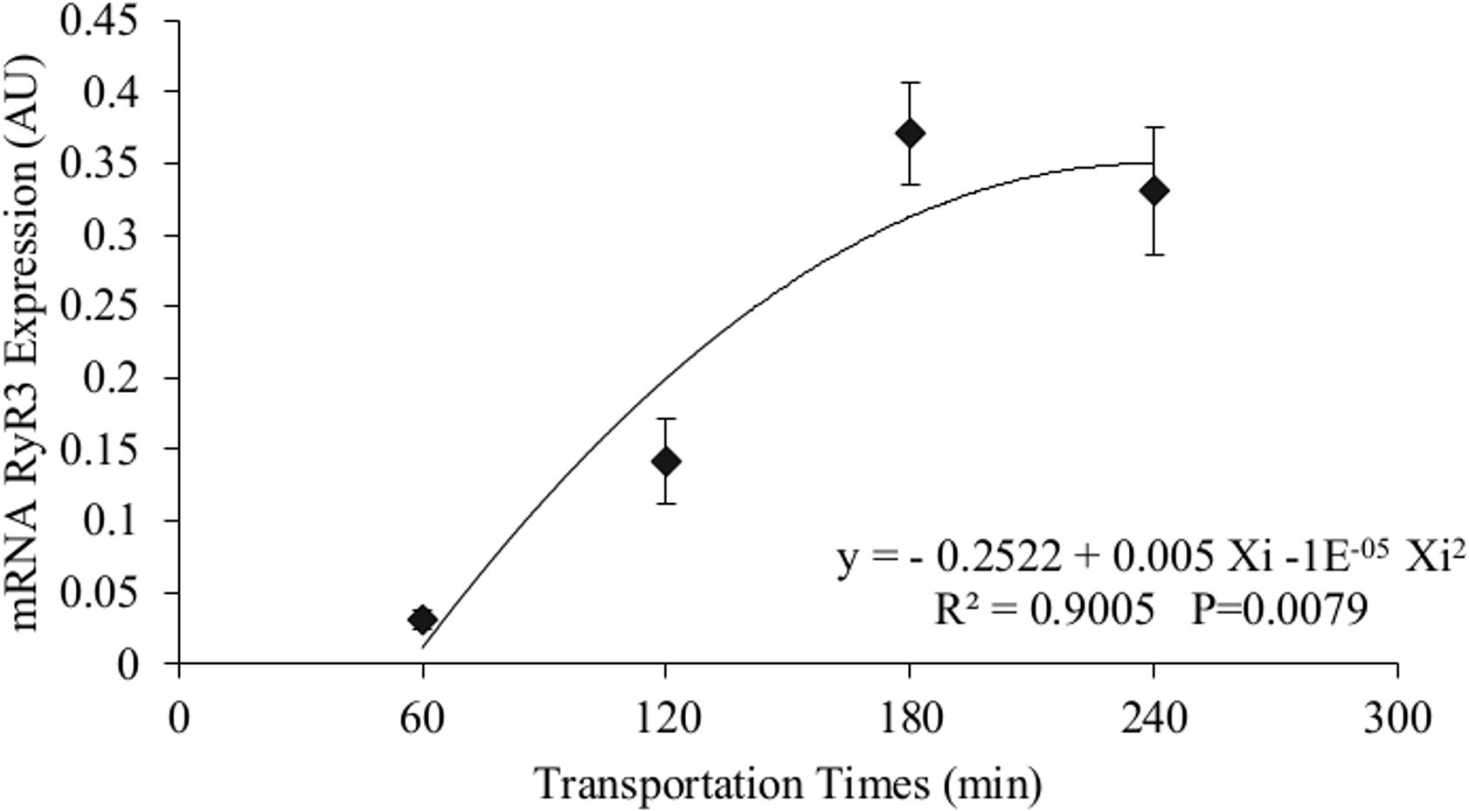
Protein receptor ryanodine type 3 gene expression in muscle. mRNA RyR3 expression in Nile tilapia muscle submitted to 200 kg/m^3^ density at different transportation times (60, 120, 180, and 240 minutes). AU is abbreviation for Arbitrary Unit. Vertical lines represent the standard error of the mean between the fifteen replications per time of transportation.

A significant interaction between density and time factors (P = 0.004) was observed when type 3 mRNA ryanodine receptor protein (RyR3) expression was assessed. During interaction, a significant effect of regression on different transportation times was not detected for densities 100 and 400 kg/m^3^; however, a quadratic effect could be perceived at 200 kg/m^3^ density (y = -0.2522 + 0.005Xi -1E^-05^Xi^2^, R^2^ = 0.90), where the lowest mRNA RyR3 expression was reported at 60 minutes of transportation (0.031 AU) and the highest value at 180 minutes (0.371 AU) (Fig 4).

**Fig 5:**
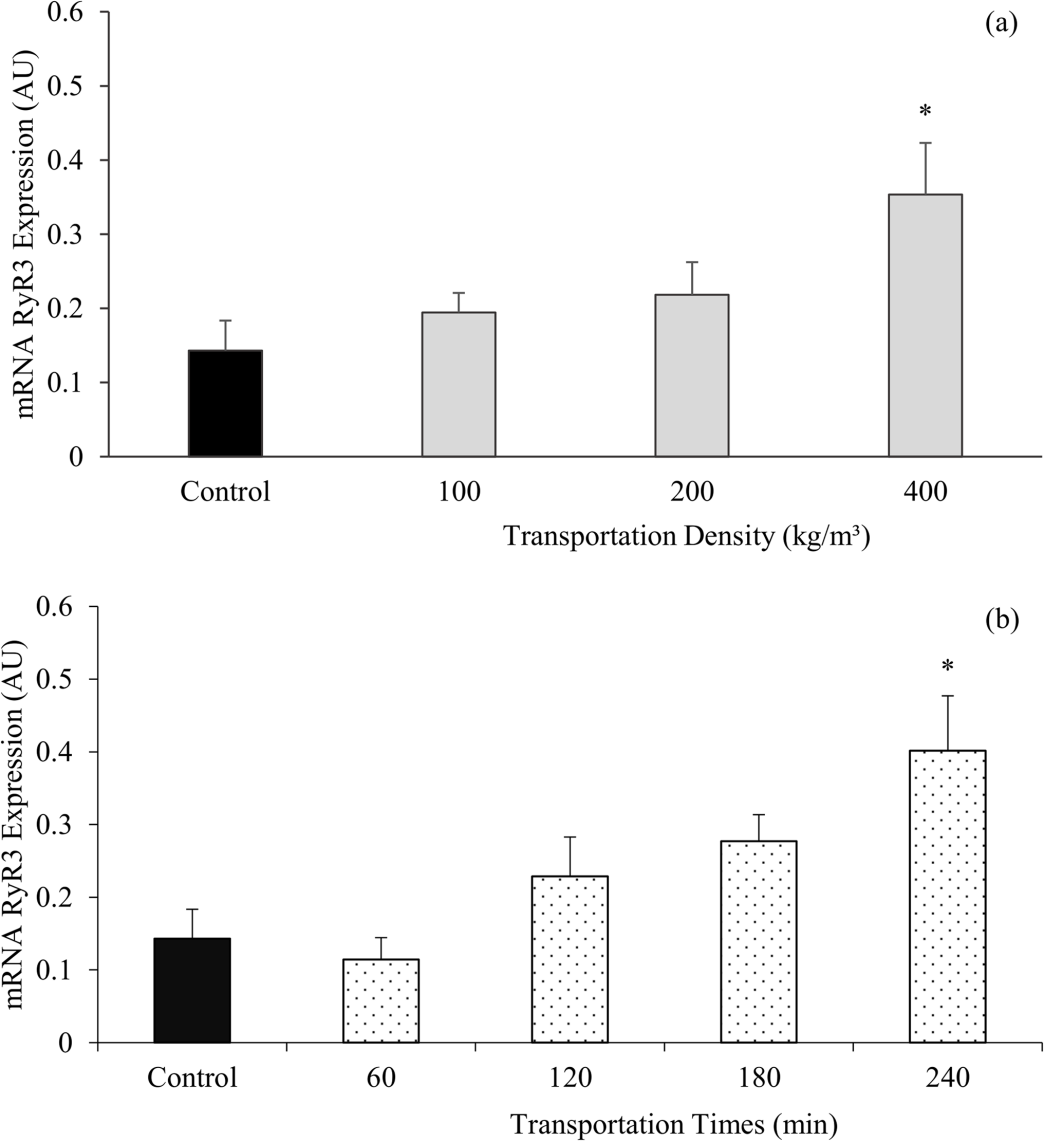
Protein receptor ryanodine type 1 gene expression in muscle. Nile tilapia mRNA RyR1 expression submitted to different transportation densities (100, 200, and 400 kg/m^3^) (a) and different transportation times (60, 120, 180, and 240 minutes) (b). (*) indicates a difference in treatments from the control based on Dunnett's test (P<0.05). AU is abbreviation for Arbitrary Unit. Vertical bars represent the standard error of the mean between the twenty replications (Fig 5a) per transportation density, and fifteen replications (Fig 5b) per time of transportation (control is n = 5).

Comparison of means for different transportation times with control treatment revealed a significant difference (P<0.05) only at 240 min, which showed a higher mRNA RyR3 average (0.401 AU) when compared to control treatment (0.143 AU) (Fig 5A). Fish from the 400kg/m^3^ density had the highest average (0.353 AU) when mRNA RyR3 averages of different transportation densities were compared to control (Fig 5B).

## Discussion

Fish capture, handling, and transportation are traumatic procedures that may cause severe physiological reactions, such as increased muscle activity, muscle and liver energy reserve mobilization and alterations in the acid-base blood balance [17]. In fish, the most commonly used plasmatic cortisol is the stress indicator since stress increases cortisol levels [18]. In current study, soon after capture, high cortisol rates were noted until 60 minutes of transportation. As transportation time elapsed to 180 minutes, a decreased concentration was detected and evidenced adaptation to transport conditions. According to Poli [19], cortisol plasmatic levels quickly increase after exposure to acute stress, and standard conditions are restored in a few hours. This demonstrates that fish have a great capacity to acclimate to new environmental conditions, albeit with reduced performance. Some degree of stress appears to have been experienced also by the fish in the control group due to handling, possibly combined with increased swimming activity prior to slaughter. This observation was also made by Hultmann et al. [20] for the Atlantic cod (*Gadus morhua*).

Vigorous swimming leads to the intense usage of the white muscle, an increase in anaerobic glycolysis and lactic acid production, a decrease in muscle pH [2, 3, 4]. Higher pH rates (6.20) were observed in fish from treatments that had lower cortisol concentrations, such as treatments 100 kg/m^3^ + 120 min (16.62 μg/dL) and 400 kg/m^3^ + 180 min (12.92 μg/dL). Thus less stressed animals apparently produced flesh with higher pH when compared to others, although this fact cannot be confirmed with isolated transportation time which significantly characterized the stress. The rapid decrease in postmortem pH is a classic indicator of stress before slaughter in many species such as pigs and poultry. It has also been reported in fish, or rather, low initial postmortem pH is associated with high stress ante-mortem [21], described by numerous authors for salmon [22, 23], eel [24, 25] and gilt-head bream [26].

Greatly stressed fish may develop high luminous flesh due to a decrease in the level of soluble muscle proteins, when these are compared to slaughtered non-stressed animals [27]. However, no relationship was found in current experiment between transportation stress and luminosity in the Nile tilapia fillets. Fillets of Atlantic cod, subjected to stress on exposure to air, developed higher luminosity when compared to those slaughtered without any stress [28]. Stress from electricity in the rainbow trout increased flesh luminosity when compared to anesthetized fish [27]. Likewise, fillets of the common carp (*Cyprinus carpio*) under narcosis with CO_2_ manifested greater luminosity when compared to fish treated with anesthetic, water and ice, and asphyxia [4].

Highest chroma b* mean (8.05) was recorded for density 400 kg/m^3^. In absolute values, this density was also observed for the highest average serum cortisol. Although transportation time has not affected chroma b*, apparently the density was able to promote change in color of fillets.

The water holding capacity (WHC), the meat´s capacity to retain water during external forces, is highly important for commercial value and consumers´ acceptance, since it is related to succulence [29, 30]. Transportation time significantly affected WHC in current assay. The parameter increased as transportation time increased, ranging between 57.76% at 60 minutes and 59.01% at 240 minutes. Since the highest serum cortisol rates were observed at 60 minutes transportation time, it may be inferred that stressed fish developed meat with less WHC. The parameter increased as stress decreased. The Atlantic cod subjected to stress before slaughter developed meat with low water-holding capacity when compared to that in non-stressed fish [20].

Lund et al. [29] reported that the muscle capacity to retain water is affected by many factors, such as pH, postmortem protein oxidation, proteolytic activity of meat tenderizer enzymes, and cross-linking of myofibrillar proteins. On the other hand, low proteolytic activity may negatively influence WHC and meat succulence [6].

The genetic factors linked to changes in muscle WHC are related to alterations in the activity of ryanodine receptors (RyR) which may decrease the capacity to control calcium release to the muscle´s cell cytoplasm, especially under physical stress periods [6]. The excess calcium in the cytosol causes rapid muscular contractions, consequently speeding anaerobic metabolism and post-mortem glycolysis, which drastically decreases muscular pH. Consequently, the muscle capacity to retain water contained in intracellular storage is affected.

In current study, the RyR1 gene expression at different transportation times provided similar responses to those observed in WHC. If the fish were at a high stress level in 60 minutes of transportation time, it was expected that the RyR1 gene expression would be lower. Increasing transportation time, and thus a decrease in stress, led to a greater RyR1 activity. In fact, the isoform started to regulate calcium release from the sarcoplasmic reticulum to the cytoplasm, which positively affected the muscle´s WHC. These results demonstrate for the first time the association of mRNA RyR1 expression with WHC in the Nile tilapia.

The relationship between RyR1 gene expression and animal´s stress may also be demonstrated by comparing the same expression with control treatment. The highest mRNA RyR1 expression (0.523 AU) and the lowest serum cortisol concentration (13.34 μg/dL) were registered in control.

The RyR3 gene expression was significant for the 200 kg/m^3^ density. In this case, a quadratic regression occurred. In fact, increased transportation time to 180 minutes was also accompanied by increase in mRNA RyR3 expression, with a similar behavior for RyR1, cortisol serum and WHC.

In current assay, the lowest WHC in stressed tilapias may be due to a lower mRNA RyR1 expression which, by decreasing its potential control for Ca^2+^ release from intracellular storage, produced excess Ca^2+^ ion in the cytosol, thereby causing hypermetabolism and increased muscular contraction. The above situation possibly reduced postmortem pH and protein denaturation, with higher water loss in the meat [27]. In broilers and laying chickens, the β-RyR isoform (or RyR3) was lower in meat with a low WHC (characterized as pale, soft and exudative [PSE] meat), whereas there was no difference between normal and PSE meats for α-RyR (or RyR1) gene expression [10].

The calcium release mechanism in the skeletal muscle of non-mammalians involves α-RYR isoform, which is coupled to the dihydropyridine receptor protein (DHPR) [9]. In the muscular contraction process, the muscular fiber is stimulated through the nerve (motor endplate) and generates the action potential that spreads along the membrane surface and through the transverse tubule system towards the deepest parts of the muscular fiber. The DHPR detects the membrane depolarization, alters its conformation and activates the α-RYR which releases Ca^2+^ from the sarcoplasmic reticulum [31]. On the other hand, the β-RyR is not bonded to DHPR but is situated peripherally at the sarcoplasmic reticulum tubule T junction, and its activity is differently regulated. Part of Ca^2+^ released by α-RYR binds to the cytoplasmic domain of the β-RyR duct and opens it by releasing the calcium [9].

This mechanism possibly explains the different results obtained for RyR1 and RyR3 in the present study, where RyR1 was perfectly adjusted to the fish stress condition as a direct response in WHC. Therefore, it is possible that the defect in the calcium regulation occurs in the two isoforms, but mainly in the gene that encodes RyR1.

A ryanodine receptor protein gene mutation in swine was associated with stress-susceptible animals and prone towards the development of PSE meat [32]. Since the ß-RyR (RyR3 homologous) isoform in chickens was expressed less in animals that developed PSE meat [10], these types of meat could be the result of a high Ca^2+^ cytosol availability.

Results in current assay show for the first time in fish the relationship between the expression of ryanodine receptor gene encoders, ante-mortem stress and water-holding capacity, specifically in Nile tilapia.

Results demonstrated that the increase in transportation time up to the 180 minute mark led to a decrease in stress, expressed by serum cortisol rates. Stressed fish presented lower RyR1 gene expression and a lower meat water-holding capacity. Decrease in stress led to a linear increase in these parameters until 180 minutes. Results provided the first evidence that pre-slaughter stress affects the ryanodine receptor gene expression and, consequently, the fillet´s water-holding capacity due to the increased availability of cytosolic Ca^2+^. Future prospects based on this study predict the possibility, similar to that in other animal species, the early identification of stress-prone specimens and the development of meat with low water-holding capacity.
